# Bee Venom Acupuncture for Shoulder Pain: A Literature Review of Clinical Studies

**DOI:** 10.3390/toxins16110501

**Published:** 2024-11-20

**Authors:** Hyein Jeong, Soobin Jang, Jang-Kyung Park, Kyeong Han Kim, Jong Hyun Park, Gihyun Lee, Soo-Hyun Sung

**Affiliations:** 1 Department of Preventive Medicine, College of Korean Medicine, Kyung Hee University, Seoul 02447, Republic of Korea; frogcream@gmail.com; 2 Department of Preventive Medicine, College of Korean Medicine, Daegu Haany University, 1 Haanydaero, Gyeongsan 38610, Republic of Korea; suebin@nate.com; 3 Department of Korean Medicine Obstetrics and Gynecology, School of Korean Medicine, Pusan National University, Yangsan 50612, Republic of Korea; vivat314@pusan.ac.kr; 4 Department of Preventive Medicine, College of Korean Medicine, Woosuk University, Jeonju 54986, Republic of Korea; solip922@hanmail.net; 5 Department of Pathology, College of Korean Medicine, Daegu Haany University, 1 Haanydaero, Gyeongsan 38610, Republic of Korea; moguri@dhu.ac.kr; 6 College of Korean Medicine, Dongshin University, Naju 58245, Republic of Korea; 7 Department of Policy Development, National Institute for Korean Medicine Development, Seoul 04516, Republic of Korea

**Keywords:** bee venom, bee venom acupuncture, shoulder pain, clinical studies

## Abstract

Managing shoulder pain typically involves the use of acetaminophen or oral nonsteroidal anti-inflammatory drugs, but prolonged use of these medications can lead to dependence and various side effects. To overcome the dose dependency and side effects of these conventional drugs, animal venoms have begun to be utilized. Among them, bee venom stands out for its powerful anti-inflammatory properties, which help relieve pain and treat chronic inflammatory conditions. This review evaluates the efficacy and safety of bee venom acupuncture (BVA) for shoulder pain. In March 2024, we searched 11 databases: 5 international and 6 Korean databases. We identified 23 clinical studies on BVA for shoulder pain. The causes of shoulder pain were post-stroke pain (43.5%), rotator cuff syndrome (17.4%), and brachial plexus palsy (13.0%). The BVA concentration and dosage per session were 0.005–1.0 mg/mL and 0.01–2.0 mL, respectively. All included clinical studies reported positive effects on pain outcomes. This review suggests that BVA, which involves injecting bee venom into acupuncture points, may serve as a viable alternative for pain management. However, the level of evidence in the included studies was low and adverse effects were reported infrequently, indicating that further research is needed.

## 1. Introduction

Because the shoulder joint is the most complex anatomical structure in the human body, shoulder disease can cause acute and chronic pain as well as functional disorders and weakness of the upper extremities in various ways [1,2]. Shoulder pain, along with pain in the lower back and knees, is one of the most frequently encountered musculoskeletal disorders. [2]. The 1-year prevalence of shoulder pain can reach up to 55% [3]. Based on a previous systematic review, the lifetime prevalence of shoulder pain can reach up to 67% [4]. Conventional medicine approaches the diagnosis and treatment of shoulder diseases by managing shoulder pain using acetaminophen or oral nonsteroidal anti-inflammatory drugs (NSAIDs) [5]. Although managing shoulder pain with acetaminophen or oral NSAIDs is effective for pain relief, various side effects may occur [5]. Therefore, further research is needed on treatment options that can control pain while addressing the underlying causes of shoulder disorders.

Acetaminophen and NSAIDs are drugs that have been used worldwide for a long time to reduce pain and inflammation [6,7]. However, NSAIDs have been reported to cause a range of side effects, including mild issues such as gastritis, elevated blood pressure, skin rash, hives, dizziness, and headaches, as well as serious complications such as heart attack, stroke, gastrointestinal bleeding, asthma, anemia, and liver damage [6]. Acetaminophen can lead to mild side effects, such as nausea, vomiting, rashes, and hives, as well as serious issues, including asthma, liver toxicity, gastrointestinal bleeding, reduced kidney function, and blood abnormalities (e.g., thrombocytopenia and leukopenia). Additionally, acetaminophen increases the risk of bleeding when used with anticoagulants [7]. Thus, although both drugs are effective for pain control, their prolonged use can lead to dose dependence and a range of mild to severe side effects.

To address the issues of dose dependency and side effects associated with conventional drugs, researchers have investigated various strategies, especially the use of natural toxins. These are toxic substances generated by bacteria, insects, animals, or plants [8]. Mixtures of toxins that have coevolved with effective delivery systems, such as glands and injection mechanisms, are referred to as ‘venoms’ [9]. While these venoms can induce pain, illness, and even death, they also represent a valuable resource for humans in the development of new drugs aimed at addressing various conditions, ranging from pain relief to the treatment of serious diseases [8]. Recently, extensive research, ranging from experimental to clinical studies, has been conducted on animal venoms, with a particular focus on pain-related conditions. Studies have investigated the potential of various animal venoms for pain management, including those from bees [10], scorpions [11], spiders [11], snakes [12], centipedes [9], and snails [13]. These natural toxins, such as animal venoms, could serve as alternatives to acetaminophen and NSAIDs for pain control.

Bee venom (BV) is a complex blend of toxic substances that includes melittin, the mast-cell-degranulating peptide, adolapin, apamin, enzymes such as phospholipase A_2_, biologically active amines like histamine and epinephrine, and nonpeptide components, all of which exhibit various pharmaceutical properties [14]. Melittin, the main component of apitoxin, makes up 40–60% of BV’s total dry weight and triggers cell death by disrupting cell membranes through pore formation, causing hemolysis and general cytotoxicity. Despite these harmful effects, BV and its constituents have shown potential in alleviating symptoms of neurodegenerative disorders like Parkinson’s disease (PD), Alzheimer’s disease (AD), and amyotrophic lateral sclerosis (ALS), with increasing evidence supporting their neuroprotective and anti-inflammatory properties [15]. BV acupuncture (BVA) involves injecting diluted BV into acupoints; it is used to relieve pain and treat chronic inflammatory diseases, and is actively applied to shoulder and joint treatment [15,16]. In South Korea, approximately 29.9% of approximately 15,000 Traditional Korean Medicine (TKM) clinics use herbal acupuncture, with BVA being the most commonly used herbal acupuncture treatment [17,18].

Our objective was to identify clinical studies, including case studies, case–controlled trials, and randomized controlled trials, focused on BVA for treating shoulder pain, as well as to offer comprehensive information on the toxins found in bee venom.

## 2. Results

### 2.1. Study Description

**Figure 1:**
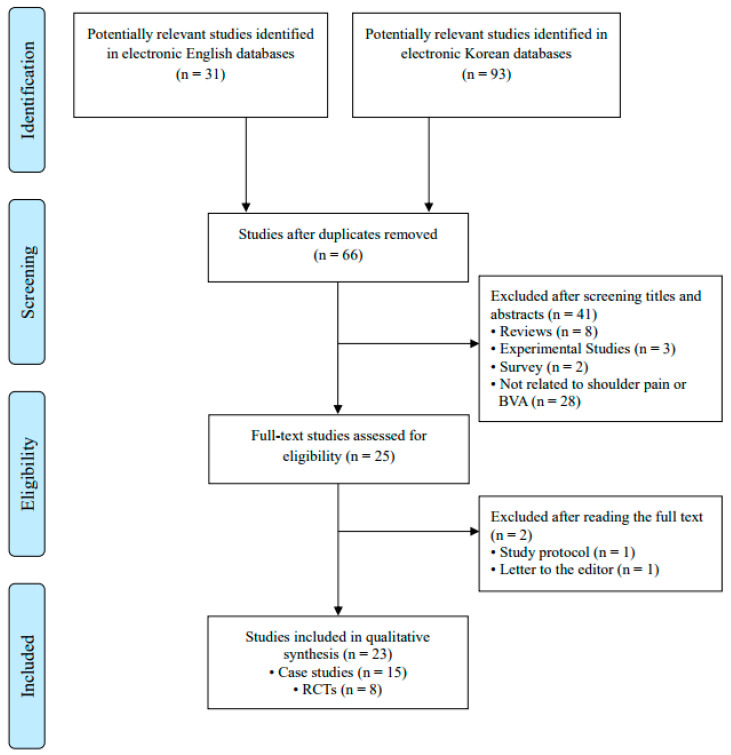
Flowchart of study selection process. BVA: bee venom acupuncture; RCTs: randomized controlled trials.

**Figure 2:**
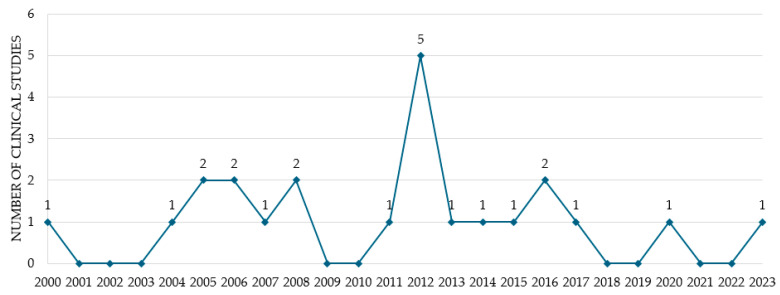
Trend of clinical trials of bee venom acupuncture on shoulder pain by year.

The database searches identified a total of 124 potentially related articles. Among these, 58 duplicate articles and 41 articles unrelated to BVA or pain were excluded. After reviewing the titles and abstracts, a total of 25 studies were considered for full article assessment. Of these, 23 studies were selected for final review, excluding one study protocol and one letter to the editor. Figure 1 presents a flowchart of the study selection process and Table 1 summarizes the specifics of the included clinical studies [19,20,21,22,23,24,25,26,27,28,29,30,31,32,33,34,35,36,37,38,39,40,41] on BVA for shoulder pain. Most studies (22 of 23, 95.7%) were conducted in South Korea, whereas 1 study was conducted in Vietnam. The initial clinical trial investigating BVA for shoulder pain in Korea was released in 2000. From 2001 to 2023, the number of trials ranged from zero to a maximum of five each year, with no clinical trials conducted in 2001, 2002, 2003, 2009, 2010, 2018, 2019, 2021, or 2022 (Figure 2). This report included 23 studies, of which 15 were case studies (65.2%) and 8 were randomized controlled trials (RCTs) (34.8%).

### 2.2. Medical Conditions and Sample Size

In the 23 studies analyzed, a total of eight distinct types of medical conditions were identified. The medical conditions reported in 19 studies included post-stroke patients with shoulder pain (43.5%), rotator cuff syndrome patients with shoulder pain (17.4%), brachial plexus palsy patients with shoulder pain (13.0%), and adhesive capsulitis patients with shoulder pain (8.7%). These conditions were all noted in more than two studies (Table 2). A total of 452 patients with shoulder pain from the 23 studies were included. The studies included in this review had sample sizes varying from 1 to 120 patients. The number of articles and patient counts according to medical conditions are shown in Table 2.

### 2.3. BVA Treatment

In all the included studies, BVA was administered through injection, indicating that practitioners injected BV into the acupoints with a syringe. The concentration of BVA used for patients with various shoulder pain conditions ranged from 0.005 to 1.0 mg/mL. Additionally, the amount of BV administered per session varied from 0.01 to 2.0 mL, while the total volume used throughout the entire treatment ranged from 0.4 to 22.8 mL (Table 3). Table 3 presents the BVA concentrations and dosages for the medical conditions of the patients, including post-stroke, rotator cuff syndrome, brachial plexus palsy, and adhesive capsulitis. BVA concentrations were not mentioned in two studies, while four studies failed to report the dosage for a single treatment, and six studies did not specify the total dosage.

### 2.4. Outcome Measures

Across the 23 clinical studies, 17 different outcome measures were utilized (see Table 4). The outcomes were classified into three categories: “statistically improved”, “improved”, and “not improved”. The visual analog scale (VAS) was the primary instrument for assessing shoulder pain severity (n = 18), with seven studies showing statistically improved outcomes and eleven showing improved outcomes. All outcome tools reported “improved” or “statistically improved” outcomes, except for the “Pain threshold measured by pressure algometer”, which reported “not improved” in one study. To assess statistical significance (statistical improvement), Case and retrospective studies were compared before and after treatment. Furthermore, the CCTs and RCTs examined the BVA and control groups for statistical significance regarding improvement.

## 3. Discussion

Analgesic drugs are effective and primarily administered orally; however, they carry a significant risk of side effects [42]. Additionally, taking multiple oral medications can cause side effects due to drug interactions [42]. Therefore, research and development of non-oral routes for pain control are necessary. BVA is a non-oral treatment method that involves the direct injection of BV into the pain site to improve pain and inflammation. Our literature review suggests that there is scientific evidence supporting the efficacy of BVA in shoulder pain and function. Although this study reviewed various conditions associated with shoulder pain and BVA concentrations and dosages, considerable variability was observed. To include BVA in evidence-based clinical practice guidelines in the future, well-designed clinical studies of high quality are needed.

As most studies did not report adverse effects (22 out of 23), it was difficult to assess the safety of BVA for shoulder pain. BVA uses purified BV diluted in distilled water [43] rather than administering live bee stings. Therefore, BVA is expected to have fewer adverse effects, such as allergic and immune reactions, than live bee stings. Kim et al. [44] reported that 16.7% of the participants in the experimental group experienced adverse effects. These symptoms ranged from mild, including localized pain, redness, swelling, and numbness, to severe symptoms such as hyperventilation and constricting chest pain. As these adverse effects are related to individual sensitivity to BV, pretreatment skin testing is recommended. Additionally, the potential toxicity of BV on normal non-target cells is an important consideration when evaluating its therapeutic applications. BV, particularly its primary component melittin, can disrupt the integrity of cell membranes, causing lysis and death in both normal and target cells by forming pores in the lipid bilayers [14,45], posing a threat to healthy tissues due to their non-specific cytotoxicity. Therefore, it is crucial that future research carefully examines and monitors these cytotoxic effects to ensure the safe use of BV in clinical applications [15].

Pain is a subjective sensation experienced by patients and is typically measured using self-reported evaluation tools, such as the visual analog scale (VAS) and numerical rating scale (NRS). While BVA is effective in improving pain, few studies have statistically validated its effectiveness compared with control groups, indicating the need for cautious interpretation. Chronic pain can affect mood, anxiety, and sleep quality, thereby decreasing overall quality of life. Although the included randomized controlled trials (RCTs) did not report mental health issues, such as depression and anxiety, or quality of life in patients with shoulder pain, high-quality RCTs are needed to investigate these outcomes further.

Among the studies included in this review, 18 studies that evaluated joint range of motion reported that BVA improved this metric. Inflammatory conditions of the shoulder, such as frozen shoulder, rotator cuff tendinitis, shoulder arthritis, calcific tendinitis, adhesive capsulitis, and bursitis, are associated with pain and limitations in shoulder range of motion [46]. Additionally, shoulder dislocation or external trauma can lead to shoulder inflammation, resulting in functional limitations and pain [46]. BVA has been reported to have anti-inflammatory and analgesic effects [47]. Therefore, it is a treatment option that not only improves shoulder pain, but also addresses both the underlying condition and pain simultaneously. Further research is needed to integrate this treatment into clinical practice guidelines for shoulder disorders.

Combining BV with prescription drugs such as acetaminophen and NSAIDs may offer synergistic benefits in pain management. Studies on chronic neck pain [48] and low back pain [49] have shown promising results when BVA is used alongside conventional medications, highlighting the potential benefits of this combination. Studies suggest that BV works through pathways involving both the nervous and immune systems, which might complement the mechanisms of acetaminophen and NSAIDs [50,51]. Thus, combining BV with these drugs could enhance therapeutic outcomes while potentially reducing the required doses of each, thereby minimizing side effects. Further studies are essential to confirm the safety and long-term efficacy of this combined treatment for various musculoskeletal conditions, including shoulder pain.

In South Korea, the government is developing Clinical Practice Guidelines (CPGs) for Traditional Korean Medicine (TKM), and a total of 45 CPGs were developed by 2024 [52]. The TKM CPG recommends BVA treatment for shoulder pain along with the following 13 conditions/symptoms: ankle sprain, non-specific chronic low back pain, knee osteoarthritis, stroke, traffic injuries, temporomandibular joint disorder, neck pain, herniated lumbar disc, gout, facial palsy, tension-type headache, cancer-related symptoms, and breast cancer.

This review has several limitations.

1. The absence of adverse effect reports makes it difficult to assess the safety of BVA. To verify the adverse events associated with BVA, large-scale RCTs are needed; however, these require significant participant recruitment and substantial research funding. Therefore, one approach to consider is to collect and analyze real-world data from primary care clinics that perform BVA to evaluate the adverse events associated with this treatment.

2. Of the 23 trials, 15 were case studies with low levels of evidence. Therefore, careful interpretation of the research results is required, and future studies should include higher-level evidence from well-conducted RCTs. BVA involves injecting BV with a syringe, whereas the control group can be set up with injections of normal saline. Thus, BVA is an intervention that allows for the blinding of both the practitioner and evaluator, making it feasible to conduct high-quality RCTs.

3. We searched electronic databases in English and Korean using systematic review search methods. However, databases in languages other than English and Korean were not included in our search.

4. Although most studies reported that BVA improved shoulder pain, the number of studies reporting statistical significance was limited. To demonstrate clinical effectiveness, the experimental group must show statistical significance compared with the sham or no-intervention groups. Additionally, to serve as a replacement for existing treatments, the experimental group must show statistical significance compared with the conventional treatment group. Future clinical research should consider the findings and limitations of this review to inform its design and implementation.

## 4. Conclusions

Clinical evidence suggests that BVA has significant potential as a non-oral treatment option for shoulder pain. The analgesic and anti-inflammatory effects of BVA enable it to simultaneously improve pain and function. To recommend BVA in the clinical field, standardized pain treatment protocols for specific shoulder conditions need to be developed. Future studies should be multicenter trials or larger prospective studies to assess the effects, tolerability, and safety profile of BVA on shoulder pain.

## 5. Materials and Methods

### 5.1. Data Sources and Searches

We searched PubMed, MEDLINE, EMBASE, Cochrane Central Register of Controlled Trials, CINAHL Plus electronic databases, and six Korean electronic databases (KoreaMed, RISS, National Library of Korea, Korea Institute of Science and Technology Information, OASIS, and Korean Traditional Knowledge Portal) for articles published until March 2024. There were no language restrictions in the article selection.

The terms used for the search were as follows: “bee venom OR bee toxin OR apitherapy OR bee venom therapy OR bee venom acupuncture” AND “shoulder pain” AND “randomized clinical studies OR clinical studies OR randomized clinical trial OR case controlled studies OR case controlled trials OR case reports OR case series OR case studies”.

### 5.2. Study Selection

Two authors independently conducted the study selection process. We included various types of clinical studies (e.g., case reports, CCTs, retrospective analyses, and RCTs) that assessed the safety and effectiveness of BVA for treating shoulder pain. All patients with shoulder pain were eligible for inclusion, regardless of age or sex. Different BVA types and outcome measures (such as VAS, ROM, NRS, SPADI, upper extremity MMT, and adverse events) were considered in the treatment of shoulder pain. Studies that were nonclinical, such as experimental research, animal studies, reviews, and surveys, were excluded.

### 5.3. Data Extraction

Data were extracted by two independent authors using a predefined extraction form. Information from the selected studies was organized by the first author, including study design, publication year, medical conditions, sample size, interventions for both experimental and control groups (including form, dosage, concentration, and treatment sessions), outcome measures, adverse events, and main results. Any disagreements were resolved through discussions with the corresponding authors.

## Figures and Tables

**Table 1:** Characteristics of Included Clinical Studies.

Fist Author (Year) Countries	Study Design	Number of Patients	Diseases	Concentration and Dosage	Adverse Events	Outcome Measure	Main Result	Reference
Yin (2000) South Korea	Case studies	*n* = 24	Post-stroke patients with shoulder pain	1. Concentration: 0.1 mg/mL 2. 1 session: n.r. 3. Total 12 sessions: n.r.	n.r.	1. VAS for shoulder pain 2. Shoulder joint ROM 3. FMMA	1. Significant (*p* < 0.05) 2. Improved 3. Significant (*p* < 0.01)	[19]
Choi (2004) South Korea	Case studies	*n* = 1	Patients with shoulder pain	1. Concentration: n.r. 2. 1 session: n.r. 3. Total session and dosage: n.r.	n.r.	1. VAS for shoulder pain	1. Improved	[20]
Cho (2005) South Korea	RCT	*n* = 23 (BVA = 12, (C) = 11)	Post-stroke patients with shoulder pain	1. Concentration: 0.05 mg/mL 2. 1 session: 0.01–1.0 mL 3. Total 6 sessions: 2.51 mL	n.r.	1. VAS for shoulder pain 2. Shoulder joint ROM 3. MMT of the upper extremity	1. NS 2. NS 3. Significant effects in BVA, NS in (C)	[21]
Eom (2005) South Korea	RCT	*n* = 30 (BVA = 20, (C) = 10)	Post-stroke patients with shoulder pain	1. Concentration: 0.005 mg/mL 2. 1 session: 0.25 mL (1–6 visits) or 0.5 mL (7–12 visits) 3. Total 12 sessions: 4.5 mL	n.r.	1. VAS for shoulder pain 2. Shoulder joint ROM 3. FMMA 4. Modified Ashworth Scale	1. Significant (*p* < 0.05) 2. Significant (*p* < 0.05) 3. Significant (*p* < 0.05) 4. Not improved	[22]
Seo (2006) South Korea	Case studies	*n* = 1	Traumatic Subacromial Syndrome patient with shoulder pain	1. Concentration: 0.05 mg/mL 2. 1 session: 0.1–1.0 mL 3. Total 8 sessions: 4.3 mL	n.r.	1. NRS for shoulder pain 2. Shoulder joint ROM	1. Improved 2. Improved	[23]
Lee (2006) South Korea	RCT	*n* = 40 (BVA = 20, (C) = 20)	Stroke sequelae patient with shoulder pain	1. Concentration: 0.1 mL or 0.25 mL 2. 1 session: 0.1–1.5 mL 3. Total 9 sessions: 0.9–13.5 mL	n.r.	1. VAS 2. Shoulder joint ROM	1. Significant (*p* < 0.05) 2. NS	[24]
Ko (2007) South Korea	Case studies	*n* = 1	Post-stroke patients with shoulder pain	1. Concentration: 0.1 mg/mL 2. 1 session: 0.6 mL 3. Total 6 sessions: 3.6 mL	n.r.	1. VAS for shoulder pain 2. PRS for shoulder pain 3. Shoulder joint ROM 4. FMMA	1. Significant (*p* < 0.05) 2. Significant (*p* < 0.05) 3. Improved 4. Improved	[25]
Lee (2008) South Korea	Case studies	*n* = 6	Post-stroke patients with shoulder pain	1. Concentration: 0.1 mg/mL 2. 1 session: 0.3–1.8 mL 3. Total 12 sessions: 6.3 mL	n.r.	1. VAS for shoulder pain 2. Shoulder joint ROM 3. Pain threshold measured by pressure algometer	1. Improved 2. Improved 3. Not improved	[26]
Han (2008) South Korea	Case studies	*n* = 2	Post-stroke patients with shoulder pain	1. Concentration: 0.1 mg/mL or 0.25 mg/mL 2. 1 session: 0.4–1.0 mL 3. Total 28–49 sessions: 2.8–6 mL (0.1 mg/mL) or 12.8–15.4 mL (0.25 mg/mL)	n.r.	1. VAS for shoulder pain 2. Physical examination 3. Shoulder joint X-ray 4. Muscular strength evaluation in compliance with a classification in AMA	1. Improved 2. Improved 3. Improved 4. Improved	[27]
Park (2011) South Korea	RCT	*n* = 40 (BVA = 21, (C) = 19)	Post-stroke hemiplegic patient with shoulder pain	1. Concentration: n.r. 2. 1 session: 0.3–0.6 mL 3. Total 12 sessions: 3.6–7.2 mL	n.r.	1. VAS for shoulder pain 2. PRS for shoulder pain 3. Shoulder joint ROM	1. Significant (*p* < 0.05) 2. Significant (*p* < 0.05) 3. NS	[28]
Cho (2012) South Korea	RCT	*n* = 22 (BVA = 11, (C) = 11)	Post-stroke patients with shoulder pain	1. Concentration: 0.1 mg/mL 2. 1 session: 0.25–0.5 mL 3. Total 12 sessions: 3–6 mL	n.r.	1. VAS for shoulder pain 2. FMMA 3. Shoulder joint ROM 4. MAS	1. significant (*p* < 0.05) 2. significant (*p* < 0.05) 3. NS 4. NS	[29]
Kwon (2012) South Korea	Case studies	*n* = 1	Intra-articular adhesive capsulitis patient with shoulder pain	1. Concentration: 0.1 mg/mL 2. 1 session: 0.1–1.0 mL 3. Total session and dosage: n.r.	n.r.	1. VAS for shoulder pain 2. Shoulder joint ROM	1. Improved 2. Improved	[30]
Park (2012) South Korea	Case studies	*n* = 1	Rotator cuff syndrome patient with shoulder pain	1. Concentration: 0.3 mg/mL 2. 1 session: 0.1 mL 3. Total 61 sessions: 6.1 mL	n.r.	1. VAS for shoulder pain 2. Shoulder joint ROM 3. Physical examination	1. Improved 2. Improved 3. Improved	[31]
Lee (2012) South Korea	Case studies	*n* = 3	Calcific tendinitis patients with shoulder pain	1. Concentration: 0.1 mg/mL 2. 1 session: n.r. 3. Total session and dosage: n.r.	n.r.	1. VAS for shoulder pain	1. Improved	[32]
Choi (2012) South Korea	Case studies	*n* = 1	Traumatic Brachial Plexus injury patient with shoulder pain	1. Concentration: 0.1 mg/mL 2. 1 session: 0.2–1.6 mL 3. Total 30 sessions: 22.8 mL	n.r.	1. VAS for shoulder pain 2. Muscular strength evaluation measured by hand grip meter	1. Improved 2. Improved	[33]
Koh (2013) South Korea	RCT	*n* = 68 (BVA1 = 22, BVA2 = 23, (C) = 23)	Post-stroke hemiplegic patient with shoulder pain	1. Concentration: 0.1 mg/mL (BVA1), 0.03 mg/mL (BVA2) 2. 1 session: 0.4–1.0 mL (0.4 mL on the first visit, 0.6 mL on the second, 0.8 mL on the third, 1.0 mL throughout the fourth to sixteenth visits) 3. Total 16 sessions: 14.8 mL	Slight pruritus, local swelling, and/or redness in 30 cases mild, generalized swelling and aching in 1 case	1. SPADI 2. VAS for shoulder pain 3. Shoulder joint ROM	1. (BVA1 vs. C) significant (*p* < 0.05) (BVA2 vs. C) NS 2. (BVA1 vs. C) significant (*p* < 0.05) (BVA2 vs. C) NS 3. NS	[34]
Park (2014) South Korea	RCT	*n* = 60 (BVA1 = 20, BVA2 = 22, (C) = 18)	Adhesive capsulitis patient with shoulder pain	1. Concentration: 0.1 mg/mL (BVA1), 0.03 mg/mL (BVA2) 2. 1 session: 0.4–1.0 mL (0.4 mL on the first visit, 0.6 mL on the second, 0.8 mL on the third, 1.0 mL throughout the fourth to sixteenth visits) 3. Total 16 sessions: 14.8 mL	n.r.	1. SPADI 2. VRS for shoulder pain	1. (BVA1 vs. C) significant (*p* < 0.05) (BVA2 vs. C) NS 2. NS	[35]
Jo (2015) South Korea	Case studies	*n* = 1	Brachial plexus palsy patient with shoulder pain	1. Concentration: 0.03 mg/mL 2. 1 session: n.r. 3. Total 66 sessions: n.r.	n.r.	1. NRS for shoulder pain 2. Shoulder joint ROM 3. MMT of the upper extremity 3. Grip strength of hand	1. Improved 2. Improved 3. Improved 4. Improved	[36]
Jeong (2016) South Korea	Case studies	*n* = 4	Rotator cuff syndrome patients with shoulder pain	1. Concentration: 0.1 mg/mL 2. 1 session:0.4–2.0 mL 3. Total 1–2 sessions:0.4–4.0 mL	None	1. NRS for shoulder pain 2. SPADI 3. Shoulder joint ROM	1. Improved 2. Improved 3. Improved	[37]
Oh (2016) South Korea	Case studies	*n* = 1	Rotator cuff syndrome patient with shoulder pain	1. Concentration: 0.3 mg/mL 2. 1 session: 0.1 mL 3. Total session and dosage: n.r.	n.r.	1. VAS for shoulder pain	1. Improved	[38]
Kim (2017) South Korea	Case studies	*n* = 3	Rotator cuff syndrome patients with shoulder pain.	1. Concentration: 0.05 mg/mL 2. 1 session: 0.05–0.1 mL 3. Total 22–29 sessions: 1.1–2.9 mL	n.r.	1. NRS for shoulder pain 2. Shoulder joint ROM	1. Improved 2. Improved	[39]
Yim (2020) South Korea	Case studies	*n* = 1	Brachial plexus palsy patient with shoulder pain	1. Concentration: 0.1 mg/mL (1,2nd), 0.25 mg/mL (3,4th) or 0.5 mg/mL (5,6th), 1 mg/mL (7–10th) 2. 1 session: 0.5–1.0 mL 3. Total 10 sessions: 5 mL (0.1 mg/mL), 2 mL (0.25 mg/mL), 2 mL (0.5 mg/mL), 4 mL (1 mg/mL)	n.r.	1. VAS for shoulder pain 2. Shoulder joint ROM 3. MMT of the upper extremity	1. Improved 2. Improved 3. Improved	[40]
Nguyen (2023) Vietnam	RCT	*n* = 120 (BVA3 = 30, BVA4 = 30, BVA5 = 30, (C) = 30)	Periarthritis humeroscapularis patients with shoulder pain	1. Concentration: 0.01 mg/mL (BVA3), 0.005 mg/mL (BVA4), 0.0025 mg/mL (BVA5) 2. 1 session: 1.2 mL 3. Total 15 sessions: 18.0 mL	n.r.	1. VAS for shoulder pain 2. Shoulder joint ROM 3. IL-10 4. IL-1β 5. TNF-α	1. (BVA5 vs. BVA3, BVA4 and C) significant (*p* < 0.01) (BVA3 vs. BVA4 and C, BVA4 vs. C) NS 2. (BVA5 vs. BVA3, BVA4 and C) significant (*p* < 0.01) (BVA3 vs. BVA4 and C, BVA4 vs. C) NS 3. (BVA5 vs. BVA3, BVA4 and C) significant (*p* < 0.01) 4. NS 5. (BVA5 vs. BVA3, BVA4 and C) significant (*p* < 0.05) (BVA3 vs. BVA4 and C, BVA4 vs. C) NS	[41]

AMA: American Medical Association; BVA: bee venom acupuncture; (C): control group; (E): BVA group; FMMA: Fugl–Meyer motor assessment; IL: interleukin; MAS: Modified Ashworth Scale; MMT: manual muscle test; n.r.: not reported; NRS: numeral rating scale; NS: no significant difference between groups or before/after intervention; PRS: pain relief scale; RCT: randomized controlled trial; ROM: range of motion; SPADI: shoulder pain and disability index; TNF: tumor necrosis factor; VAS: visual analog scale; VRS: verbal rating scale.

**Table 2:** Numbers of studies and patients according to medical conditions.

Medical Conditions	Number of Studies (%)	Total Number of Patients (Range)
Post-stroke patients with shoulder pain	10 (43.5)	256 (1–68)
Rotator cuff syndrome patient with shoulder pain	4 (17.4)	9 (1–4)
Brachial plexus palsy patient with shoulder pain	3 (13.0)	3 (1)
Adhesive capsulitis patient with shoulder pain	2 (8.7)	61 (1–60)

**Table 3:** Concentrations and dosages of bee venom by patient’s medical conditions.

Medical Conditions of Patients	Concentration (mg/mL)	Dosage	
		Dosage per Session (mL)	Dosage for Entire Treatment (mL)
Post-stroke patients with shoulder pain	0.005–0.25	0.01–1.8	0.9–15.4
Rotator cuff syndrome patient with shoulder pain	0.05–0.3	0.05–2.0	0.4–6.1
Brachial plexus palsy patient with shoulder pain	0.03–1.0	0.2–1.6	2.0–22.8
Adhesive capsulitis patient with shoulder pain	0.03–0.1	0.1–1.0	14.8

**Table 4:** Outcomes of clinical studies of bee venom acupuncture for shoulder pain.

Outcome Measures	Statistically Improved	Improved	Not Improved
VAS for shoulder pain	8 ^a^	12 ^b^	0
ROM of shoulder joint	2 ^c^	16 ^d^	0
MMT of the upper extremity	1	3 ^e^	0
NRS for shoulder pain	0	4	0
SPADI	2 ^f^	3 ^g^	0
FMMA	3	1	0
Physical examination	0	2	0
PRS	2	0	0
MAS	0	1	1
Muscular strength evaluation	0	2	0
VRS	0	1	0
Pain threshold measured by pressure algometer	0	0	1
IL-10	1	0	0
Shoulder joint X-ray	0	1	0
Grip strength of hand	0	1	0
IL-1β	0	1	0
TNF-α	1 ^h^	1 ^i^	0

^a^ (BVA5 vs. BVA4, BVA3 and C) positive b/(BVA1 vs. C) positive a; ^b^ (BVA3 vs. BVA4 and C, BVA4 vs. C) NS/(BVA2 vs. C) NS; ^c^ (BVA5 vs. BVA3, BVA4 and C) positive b; ^d^ (BVA3 vs. BVA4 and C, BVA4 vs. C) NS; ^e^ significant effects in BVA, NS in C; ^f^ (BVA1 vs. C) positive a/(BVA1 vs. C) positive a; ^g^ (BVA2 vs. C) NS/(BVA2 vs. C) NS; ^h^ (BVA5 vs. BVA3, BVA4 and C) positive a; ^i^ (BVA3 vs. BVA4 and C, BVA4 vs. C) NS. FMMA, Fugl–Meyer motor assessment; IL: interleukin, MAS: Modified Ashworth Scale; MMT, manual muscle test; NRS, numerical rating scale; PRS, pain relief scale; ROM, range of motion; SPADI: Shoulder Pain and Disability Index, TNF: tumor necrosis factor; VAS, visual analog scale; VRS: verbal rating scale.

## Data Availability

No new data were created or analyzed in this study.
